# Myocardial injury in spontaneous intracerebral hemorrhage is not predicted by prior cardiac disease or neurological status: results from the Mannheim Stroke database

**DOI:** 10.3389/fneur.2025.1510361

**Published:** 2025-02-18

**Authors:** Hendrik Lesch, Lea Haucke, Mathieu Kruska, Anne Ebert, Louisa Becker, Kristina Szabo, Ibrahim Akin, Angelika Alonso, Christian Fastner

**Affiliations:** ^1^Department of Neurology, Faculty of Medicine Mannheim, Mannheim Center for Translational Neuroscience (MCTN), University of Heidelberg, Mannheim, Germany; ^2^Department of Cardiology, Haemostaseology and Medical Intensive Care, University Medical Center Mannheim (UMM), Medical Faculty Mannheim, Heidelberg University, European Center for AngioScience (ECAS), and German Center for Cardiovascular Research (DZHK) Partner Site Heidelberg/Mannheim, Mannheim, Germany

**Keywords:** cardiac troponin, mortality, structural heart disease, stroke-induced heart injury, brain-heart interactions

## Abstract

**Background and aims:**

Elevated cardiac troponin (cTn) levels (representing myocardial injury) are frequently found in patients with spontaneous intracerebral hemorrhage (sICH). Overall, the relationship between sICH and elevated cTn levels is not well understood. The aim of this study was to investigate patient characteristics and clinical parameters in patients with sICH and myocardial injury.

**Methods:**

This is a retrospective observational study based on the Mannheim Stroke database. Consecutive patient cases with acute symptomatic sICH and available high-sensitivity cTn I (hs-cTnI) at hospital admission between 2015 and 2021 were included. Group comparisons of patient, clinical and imaging characteristics were performed between groups with and without hs-cTnI elevation. In addition, variables with suspected predictive clinical significance for hs-cTnI elevation were analyzed for their predictive value using multivariate logistic regression analysis.

**Results:**

A total of 93/498 patients with sICH (18.7%; mean age 73 ± 15 years; 51.9% females) had a hs-cTnI elevation. These patients did not have a more pronounced cerebrovascular risk profile and had a comparably low prevalence of coronary artery disease (18.5%, *p* = NS) compared to those without elevated hs-cTnI levels. Elevated hs-cTnI levels had no impact on in-hospital mortality (21.5 vs. 20.5%, *p* = NS) or functional outcome at discharge. Solely clinically relevant aortic valve stenosis, graded as moderate or higher, independently predicted hs-cTnI elevation (*p* < 0.003). Other cardiac preconditions or neurological functional parameters did not serve as significant predictors.

**Conclusions:**

Myocardial injury is common in patients with sICH. Unlike in AIS patients, elevated hs-cTnI levels were not associated with a worse functional or mortality-related in-hospital outcome. Except for clinically relevant aortic valve stenosis, structural heart disease had no significant influence as a predictor. We therefore suggest that hs-cTnI elevation in patients with sICH is related to acute myocardial damage along the brain-heart axis.

## Introduction

In patients with acute ischemic stroke (AIS) elevated levels of cardiac troponin (cTn) are routinely detected as a biomarker for myocardial injury and are strongly associated with adverse patient outcomes (1–4). Consequently, national and international guidelines recommend the routine measurement of cTn levels in patients with AIS for the purpose of further risk stratification and diagnostics following hospital admission to a specialized stroke unit (5, 6). Latest research findings indicate that myocardial injury is more often attributable to abnormalities in the brain-heart interaction rather than being primarily caused by acute myocardial infarction (AMI) with coronary atherosclerotic plaque erosion or rupture (7–9).

Even though it is estimated that between 20 and 40% of patients with spontaneous intracerebral hemorrhage (sICH) exhibit elevated cTn levels at admission, only a limited number of studies have investigated this potential association with outcome (10, 11). Hemorrhagic stroke which includes sICH and subarachnoid hemorrhage is the second most common form of stroke and by itself associated with poor patient outcomes, which may be further exacerbated by cTn elevation at admission (10–12). Although there are current clinical practice guidelines and a recently updated diagnostic algorithm to determine the primary cause of cTn elevation in AIS patients, this approach has not yet been sufficiently established for patients with sICH (13–15). In contrast to established pathomechanisms of myocardial injury in AIS patients, preliminary research suggests that the release of cTn in sICH patients is primarily due to neurocardiogenic damage in the context of an autonomic dysfunction, which subsequently results in the release of catecholamines and, consequently, adrenergic overstimulation of the heart (16). Nevertheless, there is a paucity of data concerning the definitive causes of cTn elevation and the corresponding pattern of myocardial injury in patients with sICH. Furthermore, and in sharp contrast to patients with AIS, the influence of myocardial injury and the underlying mechanisms on outcomes with sICH, has not been sufficiently investigated.

The objective of this study was to examine patient characteristics and clinical parameters associated with cTn elevation in patients with sICH.

## Materials and methods

### Ethics statement

The Ethics Committee II of the Medical Faculty Mannheim, Heidelberg University, approved the retrospective analyses of the Mannheim Stroke database (2013-813R-MA).

### Subjects

Patients were screened by retrospective review of the prospectively collected Mannheim Stroke database. Consecutive patient cases with a diagnosis of sICH and measurement of high-sensitivity cardiac Troponin I (hs-cTnI) at admission between 2015 and 2021 were identified. The inclusion criteria were as follows: (i) hospitalization with an admission diagnosis of acute symptomatic sICH, as verified by cranial computed tomography (cCT) or cranial magnetic resonance imaging (ii) age 18 years or older, and (iii) analysis of hs-cTnI levels at the time of hospital admission (17).

The exclusion criteria were as follows: (i) insufficient medical records, (ii) patients suffering from traumatic ICH, admission diagnosis of AIS as well as patients with ICH caused by one of the following entities: cerebral aneurysm, arterio-venous malformation, infection, or tumor.

### Review of medical records

The patient demographic and clinical characteristics were pseudonymized and recorded from the medical records. This included general demographic parameters such as age, vascular risk factors as well as relevant neurological and cardiac comorbidities.

The neurological patient data included the National Institutes of Health Stroke Scale score (NIHSS) at admission, after 24 h, and at discharge, as well as the modified Rankin Score (mRS) at admission and at discharge, and the mRS prior to hospitalization.

The imaging data included the location of the sICH, the presence of intraventricular hemorrhage (IVH), as well as hematoma volume and expansion on follow-up imaging, defined as an increased volume (estimation of hemorrhage volume using the established ABC/2 method) of >33% from the initial imaging. In a second step, comparison of patients with large hematoma volume, defined as a hematoma volume on initial imaging >30 mL, and elevated hs-cTnI levels was performed. Additionally, the sICH characteristics included the most probable sICH etiology [due to cerebral amyloid angiopathy, hypertension, medication with oral anticoagulants (OAC), hemorrhagic transformation in AIS, or undetermined cause of sICH].

Cardiac characteristics were recorded as follows: All hs-cTnI levels obtained at admission, results of echocardiography, including systolic left ventricular function estimated by left ventricular ejection fraction (LVEF) and valvular pathologies. All recorded hs-cTnI levels were measured in-house using SIEMENS Dimension Vista intelligent lab systems (Siemens Healthineers AG, Forchheim, Germany). All hs-cTnI levels were interpreted as either normal (hs-cTnI ≤ 0.045 μg/L), borderline elevated (hs-cTnI 0.046–0.099 μg/L), or as significantly elevated [hs-cTnI ≥0.1 μg/L; (18)]. A normal LVEF was defined as an LVEF >50%, while a reduced LVEF was defined as an LVEF ≤ 50%. Reduced LVEF was further classified as slightly impaired (LVEF 50–41%) moderately impaired (LVEF 40–30%) and severely impaired (LVEF < 30%). Known history of atrial fibrillation and coronary artery disease (CAD) were recorded. Cases with clinically significant aortic valve or mitral valve stenosis or regurgitation were grouped as significant valvular vitium. Significant valvular vitium was defined as moderate or severe stenosis or regurgitation, as diagnosed by echocardiography.

### Statistical analysis

Statistical analysis was performed by SPSS for Windows, version 29.0.2.0 (SPSS Inc., Chicago, IL, USA). For analysis of baseline characteristics, patients were initially dichotomized according to hs-cTnI levels at admission (hs-cTnI ≤ 0.045 μg/L as normal cTn levels and hs-cTnI >0.045 μg/L as elevated cTn levels). Percentages were calculated using the total number of patients for whom values for the given variable were obtained. In a second step, patients with normal hs-cTnI levels were compared to patients with borderline elevated hs-cTnI levels. The significance level was set at α = 0.05. Metric data were presented as mean with standard deviation and compared using *t*-test, non-parametric data were presented with median and interquartile range (IQR) and compared with the Mann-Whitney U test. Nominal data were compared using the chi-square or Fisher's exact test, as appropriate. For comparison among those groups, analysis of variance and the Kruskal-Wallis test were performed, depending on the mode of data distribution. All parameters showing a statistical trend in univariate analysis and clinically validated were included in a multivariate regression model to identify parameters being independently associated with hs-cTnI elevation.

## Results

### Patient characteristics

**Figure 1:**
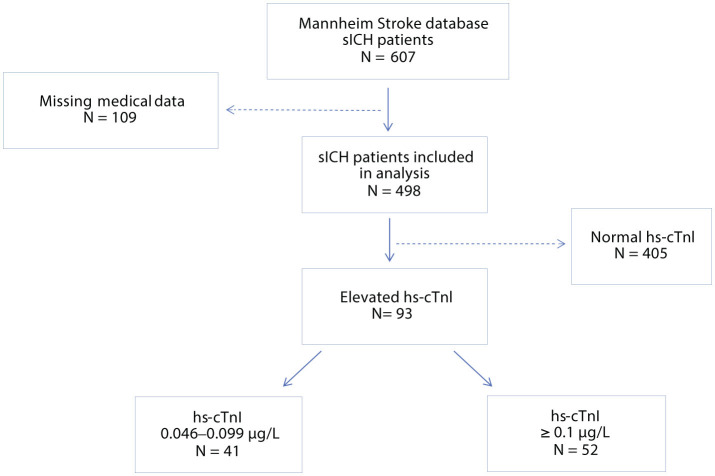
Screening procedure hs-cTnI, high-sensitivity cardiac Troponin I; N, number; sICH, spontaneous intracerebral hemorrhage.

Of the 607 patient cases with sICH, 498 patient cases met the inclusion criteria and were thus included in the analysis (Figure 1). A total of 93/498 patients (18.7%; mean age 73 ± 15 years, 51.9% females) with sICH were found to have a hs-cTnI elevation (i.e., hs-cTnI level >0.045 μg/L) at admission. There was no significant difference between patients with hs-cTnI elevation and patients without regarding baseline characteristics (Table 1). Patients with hs-cTnI elevation exhibited a comparable cerebrovascular risk profile and disease burden with regard to their prior medical history, including arterial hypertension, hypertensive heart disease, atrial fibrillation, dyslipidemia, and CAD {18.5 versus (vs.) 14.7%; [χ^2^ (1, *N* = 493) = 0.814, *p* = 0.367]}. However, patients in this group were more likely to be current smokers {19.6 vs. 7.2%; [χ^2^ (1, *N* = 493) = 13.198, *p* < 0.001]} or to have suffered a previous AMI {21.7 vs. 7.5%; [χ^2^ (1, *N* = 493) = 16.692, *p* < 0.001]}.

**Table 1:** Patient characteristics.

**Characteristics**	**Total cohort**	**Elevated hs-cTnI**	**Normal hs-cTnI**	***p*-value**
**General features**				
Number of patients, *n*	498	93 (18.7%)	405 (81.3%)	-
Age, mean ± SD	75.0 ± 13.2	73.0 ± 14.3	75.4 ± 12.9	0.10
Gender, female, *n* (%)	233 (46.8%)	43 (46.2%)	190 (46.9%)	0.91
Length (days) of hospitalization, median (IQR)	12 (6, 18)	13 (7, 21)	11 (6, 18)	0.09
**Vascular risk factors**				
Dyslipidemia, *n* (%)	84 (17.0%)	17 (18.5%)	67 (16.7%)	0.68
Alcohol, *n* (%)	30 (6.1%)	5 (5.4%)	25 (6.2%)	0.32
Smoking, *n* (%)	47 (9.5%)	18 (19.6%)	29 (7.2%)	0.001^*^
**Neurological status**				
NIHSS admission, median (IQR)	7 (3, 15)	9 (4, 19)	9 (4, 18)	0.78
NIHSS after 24 h, median (IQR)	8 (2, 16)	11 (5, 20)	10 (4, 18)	0.35
NIHSS discharge, median (IQR)	7 (2, 21)	10 (2, 26)	9 (2, 22)	0.56
Prior mRS, median (IQR)	0 (0; 1)	0 (0; 1)	0 (0; 1)	0.93
mRS admission, median (IQR)	5 (4, 5)	5 (4, 5)	5 (4, 5)	0.94
mRS discharge, median (IQR)	5 (2, 5)	5 (3, 5)	5 (3, 5)	0.94
**Cardiological features**				
Arterial hypertension, *n* (%)	427 (86.6%)	78 (84.8%)	349 (87.0%)	0.57
Hypertensive heart disease, *n* (%)	159 (58.2%)	25 (53.2%)	134 (59.3%)	0.44
Atrial fibrillation, *n* (%)	146 (29.6%)	27 (29.3%)	119 (29.7%)	0.95
Relevant CAD, *n* (%)	76 (15.4%)	17 (18.5%)	59 (14.7%)	0.37
Prior MI, *n* (%)	50 (10.1%)	20 (21.7%)	30 (7.5%)	< 0.001^*^
Sign. vavular vitium, *n* (%)	58 (20.8%)	19 (35.8%)	39 (17.3%)	0.003^*^
Sign. aortic valve stenosis, *n* (%)	17 (6.4%)	8 (16.0%)	9 (4.1%)	0.002^*^
Sign. mitral valve regurgitation, *n* (%)	28 (10.1%)	12 (22.2%)	16 (7.2%)	0.001^*^
Impaired LVEF, *n* (%)	23 (8%)	8 (14.5%)	15 (6.5%)	0.047^*^

^*^ indicates significant difference between groups; CAD, coronary artery disease; hs-cTnI, high-sensitivity cardiac Troponin I; IQR, interquartile range; LVEF, left ventricular ejection fraction; NIHSS, National Institutes of Health Stroke Scale; n, number; mRS, modified Rankin Scale; MI, myocardial infarction; Sign., significant; SD, standard deviation.

Results concerning imaging features of sICH are shown in Table 2. Overall median hematoma volume in patients with elevated hs-cTnI levels was smaller than in patients with normal hs-cTnI levels [5 mL (IQR 1.8; 19.3) vs. 9.6 mL (IQR 3; 25.925); (*U* = 15354.5; *Z* = 2.0279, *p* = 0.042); Supplementary Figure 1]. Patients with large hematoma volume, defined as >30 mL on initial imaging did not show a significant higher frequency of elevated hs-cTnI levels (*p* = 0.38). Patients with large hematoma volume had a significantly higher rate of in-hospital mortality {53.5 vs. 12.6%; [χ2 (1, *N* = 489) = 80.289, *p* < 0.001]}. No significant difference was found between location of hemorrhage, secondary hematoma enlargement or IVH between patients with elevated hs-cTnI levels and those without (Supplementary Figures 2–4). Representative examples of sICH location and IVH are shown in Supplementary Figure 5. The etiology of sICH showed no significant differences between patients with hs-cTnI elevation and those without. Patients with hs-cTnI elevation were significantly less likely to be on OAC at the time of the bleeding event {7.5 vs. 18.0%; [χ^2^ (1, *N* = 498) = 6.182, *p* = 0.013]}. In patients with sICH due to symptomatic hemorrhagic transformation of AIS, there was a higher frequency of elevated hs-cTnI levels {38.7 vs. 23.5%; [χ^2^ (1, *N* = 498) = 9.077, *p* = 0.003]}.

**Table 2:** Spontaneous intracerebral hemorrhage features.

**sICH features**	**Total cohort**	**Elevated hs-cTnI**	**Normal hs-cTnI**	***p*-value**
Lobar, *n* (%)	189 (41.0%)	31 (38.3%)	158 (41.6%)	0.72
Subcortical, *n* (%)	272 (59.0%)	50 (61.7%)	222 (58.4%)	0.78
Hematoma volume (mL), median (IQR)	9.1 (2.6; 24.55)	5 (1.8; 19.3)	9.6 (3; 25.925)	0.042^*^
Large hematoma volume (>30 mL), *n* (%)	99 (20.2%)	15 (16.9%)	84 (21.0%)	0.38
Hematoma enlargement, *n* (%)	65 (15.6%)	8 (10.1%)	57 (16.9%)	0.14
IVH, *n* (%)	135 (27.1%)	24 (25.8%)	111 (27.4%)	0.75
CAA, *n* (%)	53 (10.6%)	6 (6.5%)	47 (11.6%)	0.15
Hypertensive, *n* (%)	139 (27.9%)	29 (31.25)	110 (27.2%)	0.44
Hemorrhagic transformation, *n* (%)	131 (26.3%)	36 (38.7%)	95 (23.5%)	0.004^*^
Due to oral anticoagulants, *n* (%)	80 (16.1%)	7 (7.5%)	73 (18.0%)	0.013^*^
Undetermined etiology, *n* (%)	78 (15.7%)	12 (12.9%)	66 (16.3%)	0.42

^*^indicates significant difference between groups; CAA, cerebral amyloid angiopathy; hs-cTnI, high-sensitivity cardiac Troponin I; sICH, spontaneous intracerebral hemorrhage; IQR, interquartile range; IVH, intraventricular hemorrhage; n, number.

### Patient outcomes

Results concerning patient neurological outcomes are shown in Table 1 in section neurological status and mortality-related outcomes are shown in Table 3. Patients with sICH in both groups showed moderate neurological impairment as a result of the event, as indicated by a median NIHSS score of 9 (IQR 4;19) at admission in the group of patients with hs-cTnI elevation and 9 (IQR 4;18) in the group without (*p* = 0.78). There neither was a significant difference between the two groups regarding NIHSS after 24 h, nor regarding mRS prior to hospitalization or mRS at admission. Elevated hs-cTnI levels were not associated with overall in-hospital mortality {21.5 vs. 20.5%, [χ^2^ (1, *N* = 498) = 0.047, *p* = 0.828]} or early in-hospital mortality (i.e., within 72 h of hospitalization or within 7 days of hospitalization, respectively). The functional outcome demonstrated no significant difference between the groups at discharge [median NIHSS score 10 (IQR 2; 26) vs. 9 (IQR 2; 22); (*U* = 15633.500; *Z* = −0.588, *p* = 0.557) and median mRS 5 (IQR 3; 5) vs. 5 (IQR 3; 5); (*U* = 16667.500; *Z* = −0.070, *p* = 0.944)]. In an additional step, to analyze the association between extreme hs-cTnI levels and patient outcomes, subpopulations were created that included patients with hs-cTnI levels below the first quartile compared to patients with hs-cTnI levels above the third quartile of all hs-cTnI levels. There was no significant difference between these two groups regarding NIHSS after 24 h, at discharge or mRS at discharge (Supplementary Table 1). A hs-cTnI level in the third quartile did not lead to a significantly higher rate of in-hospital deaths compared to patients with hs-cTnI level in the first quartile.

**Table 3:** In-hospital mortality.

**Mortality**	**Total cohort**	**Elevated hs-cTnI**	**Normal hs-cTnI**	***p*-value**
Overall In-hospital mortality, *n* (%)	103 (20.7%)	20 (21.5%)	83 (20.5%)	0.83
Death within 72 h, *n* (%)	20 (19.4%)	3 (15%)	17 (20.5%)	0.58
Death within 7 days, *n* (%)	63 (61.2%)	10 (50%)	53 (63.9%)	0.25

hs-cTnI, high-sensitivity cardiac Troponin I; n, number.

### Echocardiographic data

Echocardiographic data are shown in Table 1 in section *cardiological features*. The comparison of systolic left ventricular function revealed that patients with hs-cTnI elevation exhibited a higher prevalence of reduced LVEF overall {14.5 vs. 6.5%; [χ^2^ (1, *N* = 287) = 3.937, *p* = 0.047]} and a greater likelihood of severely impaired LVEF {7.3 vs. 1.7%; [χ2 (1, *N* = 287) = 5.051, *p* = 0.025]}.

A significant valvular vitium was more frequently found in patients with hs-cTnI elevation {35.8 vs. 17.3%; [χ^2^ (1, *N* = 279) = 9.012, *p* = 0.003]}. Specifically, they exhibited a greater prevalence of significant aortic valve stenosis {16.0 vs. 4.1%; [χ^2^ (1, *N* = 267) = 9.576, *p* = 0.002]}, as well as of significant mitral valve regurgitation {22.2 vs. 7.2%; [χ^2^ (1, *N* = 276) = 10.742, *p* = 0.001]}. No significant difference was noted in the prevalence of aortic valve regurgitation between the two groups. Only one patient showed a significant mitral valve stenosis.

### Multivariate analysis

In a further step, variables with suspected clinical significance for hs-cTnI elevation were subjected to multivariate logistic regression analysis to ascertain their predictive value. Only significant aortic valve stenosis [*p* < 0.003, Exp (B) = 5.871, CI (1.85–18.66)] was identified as a predictor of elevated hs-cTnI levels. In contrast, the presence of loss of independence, as defined by mRS at admission ≥ 3, an increasing NIHSS score at admission, infratentorial bleeding location, the presence of IVH or symptomatic hemorrhagic transformation of an AIS, impaired systolic left ventricular function, CAD, or atrial fibrillation were not found to have predictive value.

### Borderline elevated hs-cTnI levels

A total of 41/498 patients (8.2%) showed borderline elevation of hs-cTnI levels (i.e., hs-cTnI 0.046–0.099 μg/L) at admission. There was no significant difference between patients with borderline hs-cTnI elevation and patients without borderline hs-cTnI elevation regarding imaging data, etiology of the sICH, neurological parameters and echocardiographic findings. Patients with borderline hs-cTnI elevation had a comparable cerebrovascular risk profile and disease burden. In patients with borderline hs-cTnI elevation, none of the clinical or imaging variables revealed significant predictive value for hs-cTnI elevation in a multivariate logistic regression analysis.

## Discussion

In the present study on a relevant population of sICH patients elevated hs-cTnI levels at admission were prevalent, consistent with prior studies on sICH patients. However, the frequency in our recent population was below the ones previously reported (10, 11, 19, 20). Baseline clinical characteristics revealed no significant differences between these patients and those without hs-cTnI elevation. A major finding of our data is that neither cardiac preconditions nor signs of structural heart disease, such as impaired LVEF or atrial fibrillation, nor neurological status were able to predict myocardial injury, with the exception of significant aortic valve stenosis. While sICH and CAD share several risk factors such as arterial hypertension, preexisting CAD in particular was relatively rare in our study population and did not predict hs-cTnI elevation.

Even though some clinical considerations suggest that elevated cTn levels in patients with sICH are associated with worse in-hospital functional and mortality-related outcome, there is still conflicting evidence on this topic, with some studies confirming this hypothesis (4, 10, 19, 21) and others failing to demonstrate this predictive value of cTn levels on outcome (22, 23). In our population, hs-cTnI elevation at admission was not associated with worse functional or mortality-related in-hospital outcomes. However, the short-term outcome of patients was poor overall with high levels of disability [median mRS at discharge 5 (IQR 3; 5) vs. 5 (IQR 3; 5)] and mortality.

While, consistent with prior studies, large hematoma volume was associated with a higher rate of in-hospital mortality, patients with large hematoma volume were not more likely to have elevated hs-cTnI levels (24, 25). Myocardial injury in sICH might therefore be triggered by more complex and interdependent mechanisms depending on bleeding location, hematoma volume, and dynamics potentiated by contributing factors such as IVH rather than hematoma volume alone.

Poor neurological status at admission may thus have contributed to the fact that hs-cTnI elevation at admission had no additional predictive influence on worse functional outcome in our population. Moreover, several other factors may have contributed to the novelty of our findings compared to previous findings:

Firstly, the inclusion criteria were heterogeneous and differed between prior studies, particularly with regard to the timing of cTn level measurement (at admission vs. within 24 h of admission vs. during hospitalization) and the cut-off values used to define elevated cTn levels. Additionally, older studies were subject to the limitation of unavailability of hs-cTnI assays (4). In the present study, all patients had hs-cTnI levels determined at the time of hospital admission and were acutely symptomatic with sICH. Previous studies did not employ a systematic approach to performing routine cTn level measurements at admission. Instead, these measurements were conducted at a later point in time, possibly introducing a selection bias for patients who were more medically unstable or more severely neurologically affected, or who were clinically suspected to suffer from AMI as the clinically derived reason for cTn level measurement, resulting in higher rates of poorer outcomes and mortality (10, 20, 26, 27). Several previous studies had a relatively small study population, with numbers ranging between 100 and 200 patients per study (20, 22, 27). In one of the earliest studies to identify an association between the prognosis of patients with sICH and the elevation of cTn levels, Hays and colleagues were compelled to exclude 37.7% of eligible patients due to the unavailability of cCT scans and an additional 15% due to the lack of cTn data (10). In this study, cTn level measurement within the first 24 h of hospitalization was subject to a significant selection bias, as patients with a history of cardiac disease or suspected AMI were more likely to receive cTn testing (10).

A further small study comprising 100 ICH patients, including those with traumatic ICH etiology, undergoing clot evacuation identified cTn elevation and the volume of hemorrhage as predictors of in-hospital mortality. In this study, cTn levels were not assessed systematically at admission: rather, they were only evaluated if specific criteria were met, such as the presence of electrocardiographic abnormalities and certain clinical parameters. Additionally, patients with a history of CAD were excluded (20). In this study, the neurological status of patients was not addressed in a systematic manner, and the short-term neurological outcomes were not compared. The interpretation of the results of this study is further complicated by the fact that surgical therapy in ICH is often restricted to young, previously healthy patients with superficial lesions and deteriorating neurological exams after admission, resulting in a severe selection bias for younger patients (20). In general, the majority of previous studies have comprised a significantly younger study population, with mean ages ranging between 50 and 60 years (19–21, 27). In comparison, the mean age of our study population was 75.0 ± 13.2. Consequently, elevated hs-cTnI levels at admission in an older study population may be indicative of overall multimorbidity, rather than neurological disability. Furthermore, at this juncture, it may not contribute significantly to neurological outcome and mortality, which are primarily caused by the initial “hit” suffered from the sICH.

In a more recent and larger prospective study, Gerner and colleagues found that peak cTn elevation at any point during hospitalization was associated with worse neurological outcome at admission and at discharge while interestingly, similar to our results, short-term mortality did not differ significantly between the groups (11). In their retrospective study, where only 63% of eligible patients with sICH had a cTn level measurement at admission, Tummala and colleagues found a higher mortality rate in patients with sICH and cTn elevation, but in a multivariate regression analysis cTn levels were no independent predictor of mortality (28).

Interestingly, in the recently published secondary observational analysis of the Factor Seven for Acute Hemorrhagic Stroke (FAST) Trial, Rosso and colleagues found increased mortality and worse neurological outcomes in patients with acute myocardial injury, defined as at least one cTn value above the upper reference limit with an increase or decrease of >20% within 48 h (21). Their data showed a very low incidence of significant cardiac complications in both patients with and without cTn elevation. In addition, no significant difference in outcomes was observed when comparing patients with cTn elevation but a decrease of cTn on subsequent measurement to patients with non-elevated cTn levels. One implication of this finding is that acute myocardial injury, rather than an isolated cTn elevation at admission, may be a potential predictor of poor outcome and mortality.

Elevated cTn levels indicate myocardial injury, but do not reveal the underlying pathology of myocardial cell damage, nor do elevated cTn levels unequivocally indicate AMI. In fact, the Fourth Universal Definition of Myocardial Infarction provides us with different types of myocardial injury, all of which are possible causes in sICH patients (29). Accordingly, myocardial injury may be caused by ischemic as well as non-ischemic pathologies or by chronic structural heart disease (29). Following the brain-heart axis, the cTn release, i.e., myocardial cell damage, can also be caused by dysregulation of the complex central autonomic network leading to enhanced stress responses, often referred to as stroke-heart syndrome as a consequence of stroke-induced heart injury (16). The central autonomic network, which includes cortical and subcortical regions and has a strong association with the insular cortex, particularly in the right hemisphere, induces responses in cardiovascular function via the autonomic nervous system and the hypothalamic-pituitary-adrenal axis (30–32). Studies have proposed that an overactivation of this network results in the release of excessive amounts of catecholamines that disrupt cardiomyocyte function, causing microcirculatory dysfunction and coronary insufficiency by vasoconstriction (13, 16). As patients with hs-cTnI elevation in the present study population did not have an overall more pronounced cerebrovascular risk profile and had a comparatively low prevalence of CAD, our findings support the growing evidence that cTn elevation in connection with sICH is primarily related to myocardial damage along the brain-heart axis and less to macrovascular ischemic conditions. However, further investigation is needed as cTn level measurement at admission may be insufficient in detecting myocardial injury. Serial cTn level measurements with the possibility to detect dynamic changes may provide a more suitable tool to identify patients suffering from acute myocardial injury.

## Limitations

The interpretation of our results is limited by the retrospective study design and the exclusion of 109 patients (18.0%) due to insufficient data. Specifically, there was no data available regarding pre-hospital delay times and therefore further analysis of its impact on in-hospital outcome could not be performed.

## Conclusions

The findings of this study indicate that patients with sICH are at an elevated risk for myocardial injury, not specifically predicted by neurological status at admission or preexisting structural heart disease. Short-term outcome might not be further influenced by hs-cTnI elevation in severely affected sICH patients. However, the etiology and predictive value of cTn elevation remain uncertain, underscoring the need for continued research to identify risk factors for myocardial injury and diagnostic algorithms to detect patients who would benefit from an intensive cardiac work-up.

## Data Availability

The raw data supporting the conclusions of this article will be made available by the authors, without undue reservation.
