# Supplementary material for: Myocardial injury in spontaneous intracerebral hemorrhage is not predicted by prior cardiac disease or neurological status: results from the Mannheim Stroke database
Source: Front Neurol. 2025 Feb 18;16:1510361. doi: 10.3389/fneur.2025.1510361 (PMC11876033; doi:10.3389/fneur.2025.1510361)
Supplement: Supplementary file 1 [file Supplementary_file_1.docx]

Supplementary Table 1 Outcomes for patients with hs-cTnI in the first vs. third quartile

| Outcome parameters | Total Subpopulation | First Quartile | Third Quartile | *p* value |
| --- | --- | --- | --- | --- |
| NIHSS after 24h, median (IQR) | 9.5 (4; 18) | 9 (3; 17) | 11 (5; 20) | 0.20 |
| NIHSS discharge, median (IQR) | 7 (2; 20) | 7 (2; 20) | 9 (2.5; 20) | 0.95 |
| mRS discharge, median (IQR) | 5 (3; 5) | 5 (3; 5) | 5 (3.75; 5) | 0.23 |
| In-hospital mortality, n (%) | 78 (20.4%) | 63 (19.6%) | 15 (25.0%) | 0.38 |

hs-cTnI, high-sensitivity cardiac Troponin I; IQR, interquartile range; n, number.

Supplementary Figure 1 Comparison of hematoma volumes (p = 0.042*)

* indicates significant difference between groups; hs-cTnI, high-sensitivity cardiac Troponin I.

Supplementary Figure 2 Comparison of percentage of patients with lobar (p = 0.72) and subcortical (p = 0.78) location of spontaneous intracerebral hemorrhage

hs-cTnI, high-sensitivity cardiac Troponin I; sICH, spontaneous intracerebral hemorrhage.

Supplementary Figure 3 Comparison of percentage of patients with hematoma enlargement (p = 0.14)

hs-cTnI, high-sensitivity cardiac Troponin I.

Supplementary Figure 4 Comparison of percentage of patients with intraventricular hemorrhage (p = 0.75)

hs-cTnI, high-sensitivity cardiac Troponin I; IVH, intraventricular hemorrhage.

Supplementary Figure 5 Representative spontaneous intracerebral hemorrhage (sICH): A, subcortical sICH; B, lobar sICH; C, intraventricular hemorrhage
